# Chronic Disease of the Knee Joint in Children

**Published:** 1891-02-21

**Authors:** 


### SURGERY.

### CHRONIC DISEASE OF THE KNEE JOINT IN CHILDREN.

There is no doubt that almost all cases of chronic joint disease, whether in children or adults, if associated with decided enlargement, are tubercular. Before the union of the epiphysis, however, still more before the commencement of ossification in the end of the bone, the type of the disease must differ from the same affection in adults. Let us for example consider the results of two common morbid processes, viz.: (1) localized tubercular synovitis, and (2) tubercular caseation of the end of the bone in a young child. In the case of the knee joint the process of ossification commences in the epiphyses of the femur and tibia very soon after birth; we have, therefore, in either of the above conditions active disease in, or in proximity to, a mass of ossifying cartilage and spongy endochondral bone. The result must be that unless promptly treated there is rapid infiltration of the tuberculous material, great swelling, and arrest—or great interference with—the growth of the bone. The looseness of the tissue would favour a diffuse lesion, but would tend to prevent actual necrosis. In the *Gazette des Hopitaux* M. Plicque contributes an article on the treatment of these affections of the knee joint in children, which he classifies under the general head of "white swelling." He enumerates and comments on the following methods of treatment:—

(1) Fixation.—Placing the joint in an immoveable apparatus, such as plaster of Paris, is one of the well-known plans. M. Plicque, however, points out that the semi-flexed position is a bad one, as the obliquity of the articular surfaces means unequal pressure. He therefore advocates the straight position. Results seem to indicate that this passive, "expectant" method, if properly persevered in, gives the most satisfactory terminations.

(2) Cauterisation.—This is best effected with the actual cautery. M. Bounet lays stress on the rapid application of *small* points. M. Richet thrusts the heated needles actually into the diseased joint. Under this heading may be noticed the classical "Scott's dressing," which appears to be considered of real value in these cases by many French surgeons.

(3) Antiseptic Injections.— These do not appear to be followed by any marked improvement. The reason of this would seem to be the impossibility of getting the fluid into actual contact with the active disease, which is not usually on the surface of the synovial membrane or articular cartilage, and which, moreover, has in most cases many centres.

(4) Resection and Erasion of the affected joint are then considered by M. Plicque, but the results of operative interference obtained by English and American surgeons are more valuable even than those of continental operators. Although the knee joint is so large and its synovial membrane so complicated, giving every opportunity for tubercular tissue to escape detection, yet the removal of the easily accessible portions of diseased membrane and bone in the operation of scraping leads to wonderfully good results. The joint may be opened either above or below the patella; the former procedure having the advantage of very freely exposing the articular ends and allowing free use of the scoop and scissors; and, moreover, the division of the muscular insertion *above* the bone being followed by quicker union, apparently, than that of the divided ligamentum patellæ.

This operation is replacing that of excision in all cases of synovial tubercular arthritis; and in those cases where the bone is deeply affected, removal of the diseased portion *only* has led to very happy results.

There is reason to believe that excision of the articular ends, although it may not immediately stop the growth of the bone, makes a considerable difference in length ultimately.

